# Patterns of Distribution of the Helminth Parasites of Freshwater Fishes of Mexico

**DOI:** 10.1371/journal.pone.0054787

**Published:** 2013-01-24

**Authors:** Benjamín Quiroz-Martínez, Guillermo Salgado-Maldonado

**Affiliations:** Universidad Nacional Autónoma de México, Instituto de Biología, Laboratorio de Helmintología, México D. F., México; Consiglio Nazionale delle Ricerche (CNR), Italy

## Abstract

In order to draw patterns in helminth parasite composition and species richness in Mexican freshwater fishes we analyse a presence-absence matrix representing every species of adult helminth parasites of freshwater fishes from 23 Mexican hydrological basins. We examine the distributional patterns of the helminth parasites with regard to the main hydrological basins of the country, and in doing so we identify areas of high diversity and point out the biotic similarities and differences among drainage basins. Our dataset allows us to evaluate the relationships among drainage basins in terms of helminth diversity. This paper shows that the helminth fauna of freshwater fishes of Mexico can characterise hydrological basins the same way as fish families do, and that the basins of south-eastern Mexico are home to a rich, predominantly Neotropical, helminth fauna whereas the basins of the Mexican Highland Plateau and the Nearctic area of Mexico harbour a less diverse Nearctic fauna, following the same pattern of distribution of their fish host families. The composition of the helminth fauna of each particular basin depends on the structure of the fish community rather than on the limnological characteristics and geographical position of the basin itself. This work shows distance decay of similarity and a clear linkage between host and parasite distributions.

## Introduction

The helminth parasite fauna of freshwater fish of Mexico ranks amongst the best characterised parasite faunas in Latin America [1] but no biogeographic synthesis of this group exists. During the past decade our knowledge of helminth parasites of freshwater fishes of Mexico has increased dramatically. More than 260 helminth species have been recorded [2], and while there is still much to do and many species remain to be described we already possess a basic understanding of its diversity. We lack, however, a summary of its distribution. Previous attempts to synthesise distributional data on a broad scale [3], [4], [5], [6], [7] are now updated as new data has become available, new taxa have been described [8], [9], [10], [11], [12], [13], [14], [15] and recent survey works have been published [16], [17], [18]. However previous studies on helminth parasites of freshwater fishes of Mexico indicate: 1) that each family has a typical helminth fauna and that species distribution corresponds to that of their hosts in such a way that the composition of the helminth fauna of a given basin is more influenced by its ichthyological composition than by limnological factors [2], [7], [19], [20]; 2) that the helminth parasite fauna of freshwater fishes of neotropical Mexico, particularly the south-eastern region is part of a Central American parasite fauna [6], [19], and that this fauna, although different, can be traced back to South American origins [19]; 3) Central American neotropical and a nearctic components have been identified within the fauna of helminth parasites of freshwater fishes in Mexico; Central American species (S = 119) parasitize typical neotropical fish families and are found mostly in Mexican neotropical drainage basins south of the Trans Mexican Volcanic Belt, 19° - 21° N meridian, while most nearctic species (S = 48) are distributed, mostly in Goodeids, from nearctic basins of Mexico in bodies of water in the Mexican Highland Plateau from the Río Lerma basin to the Río Bravo basin [21].

In this paper, we analyse the distributional patterns of adult helminth parasites of freshwater fishes of Mexico, with regard to the main hydrological basins of the country. We examine the linkage between host and parasite distributions, evaluating the relationships among drainage basins in terms of helminth diversity. Considering the points explained before, if each family of fish has a typical helminth fauna whose distribution corresponds to that of their hosts, then the ichthyological composition of the basin may be an important determinant of the patterns of distribution of the helminths. Moreover, the geographical distance amongst basins can be an important determinant of similarity in helminth faunas. Frequent contacts and exchanges of parasites between host populations of nearby basins should lead to highly homogenous faunas, we would expect the similarity in species composition among basins to decay with increasing distance between them [22], [23], [24]. We test then two hypotheses: 1) the composition of the helminth fauna of each particular basin is distributed along the two main biogeographical provinces (Nearctic and Neotropical) the same way the fish host families are distributed; and 2) the decay in compositional similarity as a function of distance separating basins. In doing so, we identify areas of high diversity and we point out the biotic similarities and differences among drainage basins. Our interest is to draw patterns in parasites composition and species richness in each basin. The fundamental postulate underlying this work is that each host family has a typical set of helminth parasites and the distribution of these helminths reflects that of the fish families they parasitize [2], [5], [7], [19], [20], [21], [25]. Each helminth species can be associated with a primary host family, even if they have been recorded in fish from different families, because records, highest prevalence and highest population densities are found in a species of a given family [2], [19], [21].

## Materials and Methods

**Figure 1:**
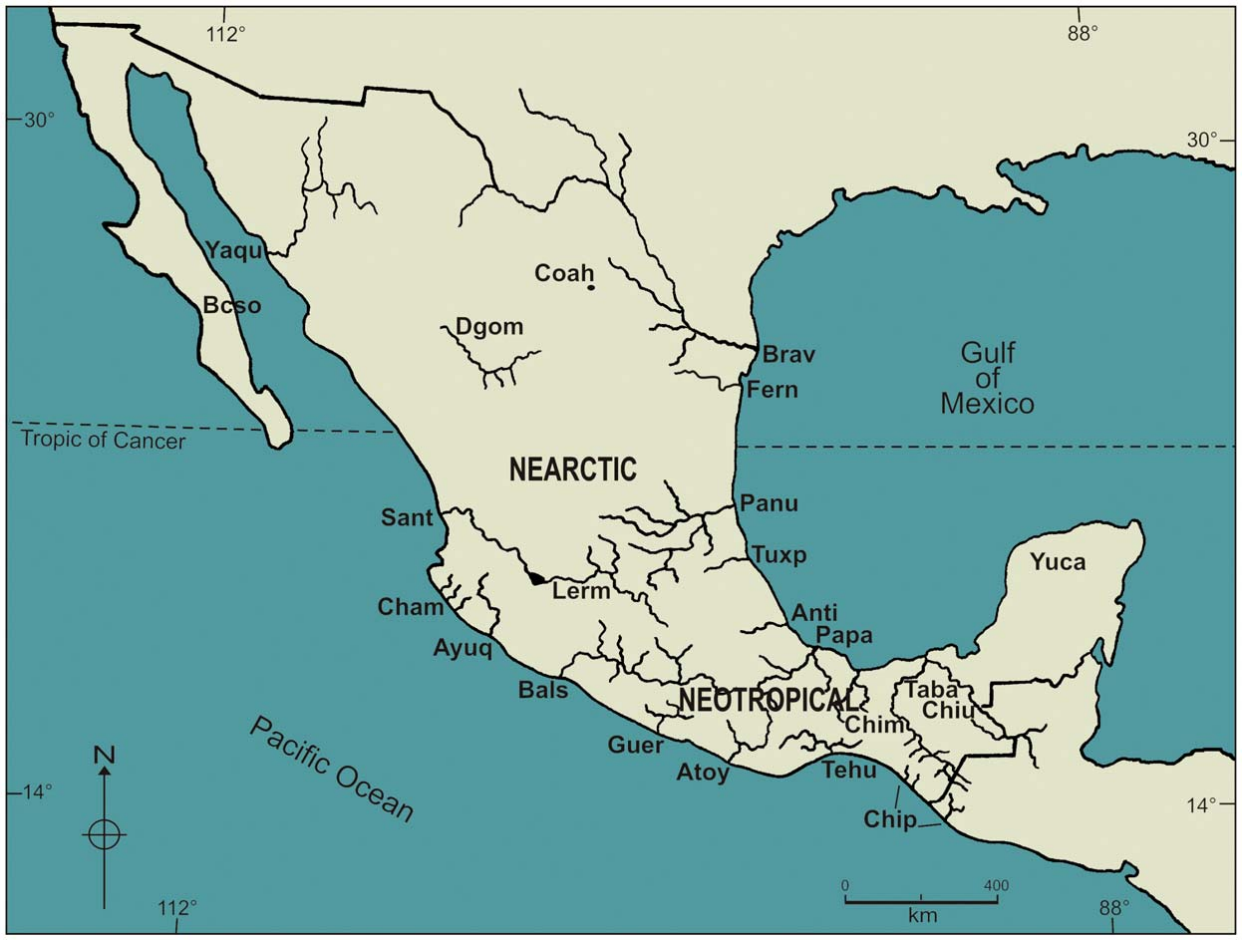
Mexican hydrological features: 1 Oases of Baja California Sur; 2 Río Yaqui; 3 rivers near Chamela, Jalisco; 4 Río Santiago; 5 Río Armería - Ayuquila; 6 Río Balsas; 7 bodies of water in Guerrero State; 8 Río Atoyac-Verde; 9 Río Tehuantepec; 10 Rivers along the south Pacific coast of Chiapas; 11 Río Bravo; 12 Río Lerma; 13 bodies of water of Valley of Cuatro Ciénegas; 14 Río Mezquital, Río Nazas and springs of Durango state; 15 Río San Fernando, Río Soto La Marina and other bodies of water in Tamaulipas; 16 Río Pánuco; 17 Río Tuxpan; 18 Río La Antigua; 19 bodies of water of Los Chimalapas; 20 Río Papaloapan; 21 bodies of water in coastal plain of Tabasco; 22 basins of Río Usumacinta and Río Grijalva, Chiapas; 23 bodies of water of the Yucatán Peninsula.

A presence-absence matrix, representing every species of adult helminth parasites of freshwater fishes from 23 Mexican hydrological basins (Fig. 1, Table 1), was compiled mainly from the extensive bibliographical research of Salgado-Maldonado [2] and from original unpublished data from our own research (i. e. we include unpublished data from Los Chimalapas, Río Tehuantepec and Río Atoyac basins in Oaxaca, from Río La Antigua, Veracruz and from Cuatro Ciénegas, Coahuila and from several bodies of water in Durango). Occasional records of some species of helminths in hosts or watersheds were excluded based in the assessment of original data in the primary literature. No records arising from theses or conference presentations are included as these are not scientific publications and most of the time the accuracy of the taxonomic identifications cannot be verified. Records of larval forms (i.e. metacercariae, metacestodes, and nematode larvae) were excluded because these larvae are dispersed by their definitive hosts, usually birds. On the other hand, freshwater fish have a limited dispersal capability and their distributional patterns result from the geological evolution of the basins they inhabit. Therefore, we are interested in adult helminths which mature and are chiefly dispersed along with its fish hosts. The records of helminths from fish in aquaculture ponds or farms [26], [27] were also excluded from the analysis as they do not represent natural populations. The helminths recorded only from primarily marine fishes in coastal lagoons, including records by Gopar-Merino *et al.* [28], Mendoza-Franco *et al.* [29] and Moravec *et al.* [30] were not considered. Introduced helminth species were included only if records of its presence concern native fish species on wild natural populations [2], [31], [32].

**Table 1:** 23 Mexican hydrological basins.

	Basin	Abbr	Latitude	Longitude	Number of species
1	Baja California	Bcso	24°54′′ N	111°17′′W	6
2	Rio Yaqui	Yaqu	not positioned		2
3	Chamela	Cham	19°23′′ N	104°58′′W	8
4	Santiago	Sant	21°46′′N	104°55′′W	10
5	Ayuquila	Ayuq	19°41′′N	104°08′′W	15
6	Balsas	Bals	18°38′′N	99°27′′W	28
7	Guerrero	Guer	19°17′′N	99°35′′W	11
8	Atoyac	Atoy	16°37′′N	96°57′′W	9
9	Tehuantepec	Tehu	16°22′′N	95°17′′W	21
10	Chiapas Pacifico	Chip	15°46′′N	93°15′′W	15
11	Rio Bravo	Brav	29°33′′N	104°23′′W	11
12	Lerma	Lerm	19°26′′N	99°54′′W	20
13	Cuatro Cienegas	Coah	26°52′′N	102°08′′W	7
14	Durango Manantiales	Dgom	24°16′′N	104°34′′W	31
15	Rio San Fernando	Fern	not positioned		3
16	Pánuco	Pánu	25°45′′N	99°23′′W	23
17	Tuxpan	Tuxp	20°55′′N	97°40′′W	5
18	Antigua	Anti	19°24′′N	97°00′′W	23
19	Chimalapas	Chim	16°53′′N	94°41′′W	27
20	Papaloapan	Papa	18°36′′N	95°39′′W	49
21	Tabasco	Taba	18°14′′N	92°49′′W	50
22	Chiapas Usumacinta	Chiu	18°05′′N	92°08′′W	51
23	Yucatán	Yuca	20°41′′N	88°11′′W	51

1 Oases of Baja California Sur; 2 Río Yaqui; 3 rivers near Chamela, Jalisco; 4 Río Santiago; 5 Río Armería - Ayuquila; 6 Río Balsas; 7 bodies of water in Guerrero State; 8 Río Atoyac-Verde; 9 Río Tehuantepec; 10 Rivers along the south Pacific coast of Chiapas; 11 Río Bravo; 12 Río Lerma; 13 bodies of water of Valley of Cuatro Ciénegas; 14 Río Mezquital, Río Nazas and springs of Durango state; 15 Río San Fernando, Río Soto La Marina and other bodies of water in Tamaulipas; 16 Río Pánuco; 17 Río Tuxpan; 18 Río La Antigua; 19 bodies of water of Los Chimalapas; 20 Río Papagayo; 21 bodies of water in coastal plain of Tabasco; 22 basins of Río Usumacinta and Río Grijalva, Chiapas; 23 bodies of water of the Yucatán Peninsula. Along with their four-letter code (abbr), geographical coordinates and total number of helminth species collected.

Major drainage basins of freshwater systems of Mexico, outlined in Fig. 1 and Table 1, were used as discrete biogeographic units and the observed species richness was compared amongst these units. The Tabasco lowlands encompass a complex net of interconnected bodies of water, channels, lakes, marshes and rivers that feed from several rivers, primarily the Grijalva and the Usumacinta river systems, the headwaters of which are located in Guatemala. However, although we acknowledge that these water sources do not constitute a single drainage basin, data from Tabasco’s water bodies, including records from Laguna de Términos, Campeche, are managed as being from a single basin. This decision was taken because the interconnections between the water bodies of this region favour free and dynamical faunal exchanges between them. The same holds for the bodies of water, mainly cenotes, located on the Yucatán peninsula. Because of insufficient data the Soto la Marina River basin, represented by three records and the Río Yaqui basin represented by two were excluded from further analysis.

Relationships between the helminthofauna inhabiting each drainage basin were examined via a similarity matrix constructed using the Sørensen coefficient. Multivariate classification is a useful method to examine biogeographic patterns exhibited by species and to distinguish and characterise biogeographic entities. In this work, drainage basins were considered the main units of comparison, and individual species occurrence represented the attributes of the individual drainage units [33], [34]. Sørensen’s similarity is, in this framework, a measure of the degree of overlap between drainages in terms of species occurrence and composition. The resulting similarity matrices were used for both cluster analysis (group average linkage) and non-metric multidimensional scaling (nMDS), as suggested by Field et al. [35] and Clarke & Warwick [36]. All multivariate analyses were undertaken using PRIMER 6 (Plymouth routines in multivariate ecological research) software package [36], [37]. Given that our aim is that of analysing the distribution of richness we performed the analysis twice, first excluding introduced helminth species and second including all introduced species. In addition, in order to evaluate the effects of geographical distance on compositional similarity (Distance Decay) we calculated the distance in km between all pairs of basins and plotted the calculated Sørensen index as a function of distance using a linear function to fit the scatter plot. We used the geographical centre or a sampled point near the geographical centre as a point of reference to calculate the distance between all pairs of basins studied. These analyses were made using Matlab software.

## Results

The compiled database includes a total of 170 species of adult helminths from 85 genera and 34 families recorded from 16 families of freshwater species of fishes in Mexico this database has been already examined for taxonomical composition and endemism by Salgado-Maldonado and Quiroz-Martínez [21]. Briefly, 119 species are neotropical, and 48 nearctic, 3 more species lack enough data to be situated. Amongst the taxa found, nematodes (S = 54 species, 31%), trematodes (S = 48, 28%) and monogeneans (S = 45, 26%) contributed 86% of the total species recorded, with cestodes (S = 14) and acanthocephalans (S = 10) being the taxa with the least recorded species. Most of the species correspond to helminth parasites of Cichlidae (S = 40 helminth species), Characidae (S = 19), Heptapteridae (S = 14), Ictaluridae (S = 13), and Poeciliidae (S = 11), 15 species of generalist helminths previously recorded from several families of freshwater fishes from North America north of Mexico, have been also recorded parasitizing fishes from Northern Mexico basins. Six introduced species have been recorded parasitizing wild native fish species in Mexico. Twenty of the 170 helminth species were recovered from 5 to 12 river basins (Table S1), these are the most widely distributed helminth species in the freshwater fishes of Mexico.The introduced Asian fish tapeworm *Bothriocephalus acheilognathi* Yamaguti, 1934 (Cestoda) that was found in 12 river basins [21].

Helminth species richness varies widely throughout drainage basins of the country (Table 1, Figure 1). Neotropical basins from south-east Mexico are rich in helminth species, whereas basins in Central Mexico and the Mexican Nearctic, north of the Eje Neovolcánico are less so. Bodies of water in the Yucatán Peninsula (S = 51), and in the Chiapas-Usumacinta basin (S = 51), as well as those grouped under the Tabasco basin (S = 50) were the richest, followed by the Papaloapan river basin (S = 49); in contrast richness observed for the rest of the studied basins ranges from S = 2 to S = 27 (Table 1).

**Figure 2:**
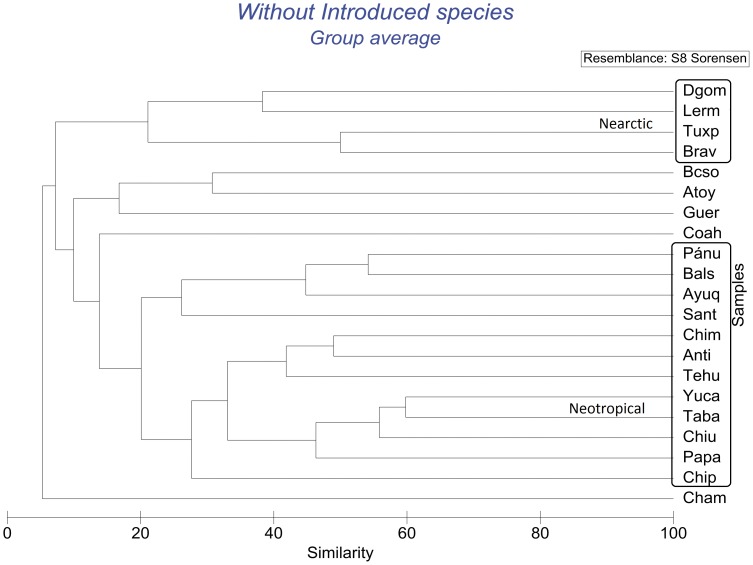
Dendrogram resulting from the similarity matrix based on the Sørensen Similarity Index for all river basins without introduced species. Nearctic and Neotropical groups are encircled, while the remaining basins correspond to the Pacific group and to the Cuatro Ciénegas river basin. Groups are based on parasite species composition of the basin.

**Figure 3:**
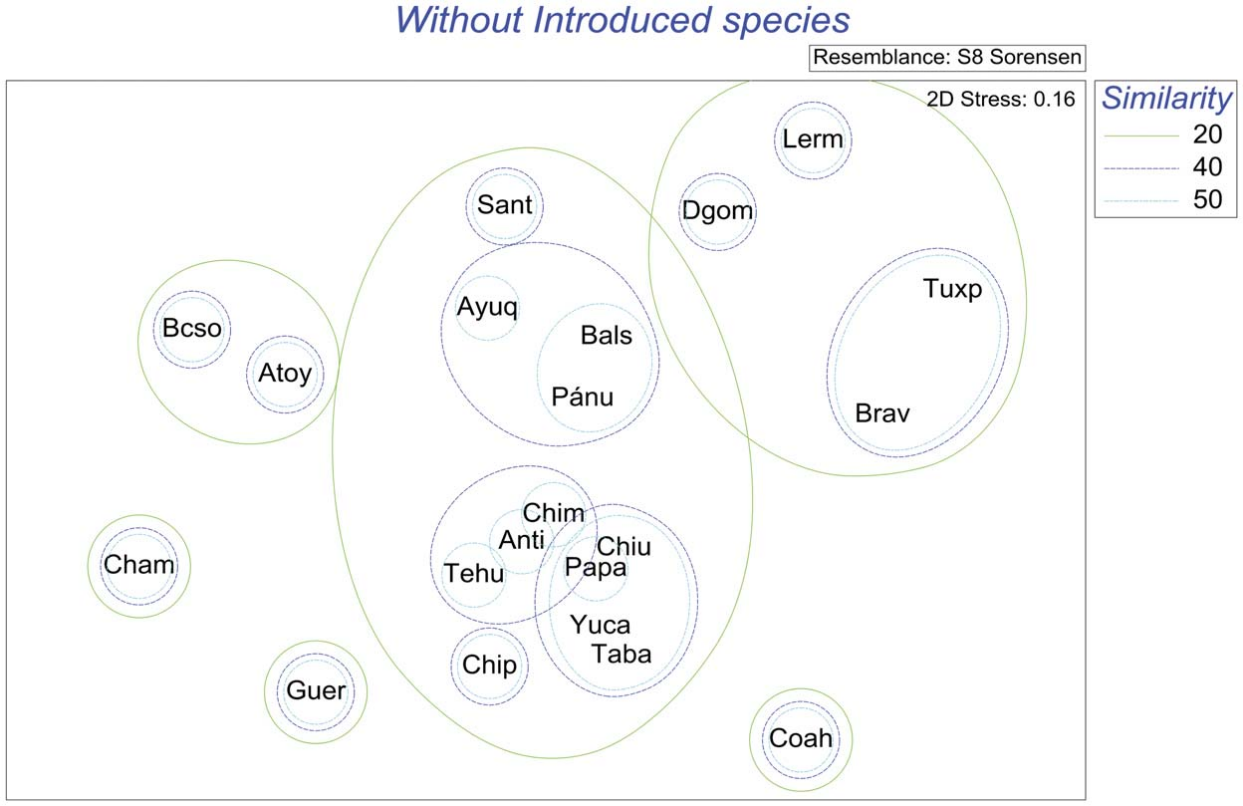
Non Metric multidimensional scaling (nMDS) ordination plot resulting from the resemblance matrix of the river basins, based on the Sørensen Similarity Index, without introduced species.

Analysis of similarity, based on Sørensen’s coefficient, of the relationships between helminth faunas of the 21 drainage basins for all species (excluding introduced species) showed three large clusters that exhibit low similarity (<10%) between them at species level. Figures 2 and 3 show the resulting dendrogram and the MDS ordination: A) A large suite of neotropical species, composed mainly of the helminth parasites recorded from Yucatán, Tabasco and Chiapas (both Usumacinta and Pacific drainages of Chiapas), plus Los Chimalapas, Río La Antigua and Río Tehuantepec faunas. To this group of neotropical species are also linked the helminths from the Balsas, Pánuco, Ayuquila and Santiago basins. B) A second group, of nearctic species includes the helminths from Rio Bravo and Río Tuxpan, plus those of the Río Lerma and from Durango. C) The third group, of Pacific affinities, includes the helminths recorded from fishes from the Oases of Baja California, the Río Atoyac-Verde, rivers near Chamela and records from Guerrero bodies of water. The fauna of helminths registered from the Valley of Cuatro Ciénegas stands alone, with neotropical links.

Results of the analyses carried out using the full species list (with the introduced species) show a somewhat disrupted pattern. The three main groups are still clearly represented in the dendrogram and the MDS ordination (Figs. S1 and S2 found in the supplemental material). However, the Río Pánuco, Balsas, Ayuquila and Río Santiago tend to group with species of nearctic affinities from Río Lerma, and Durango.

**Figure 4:**
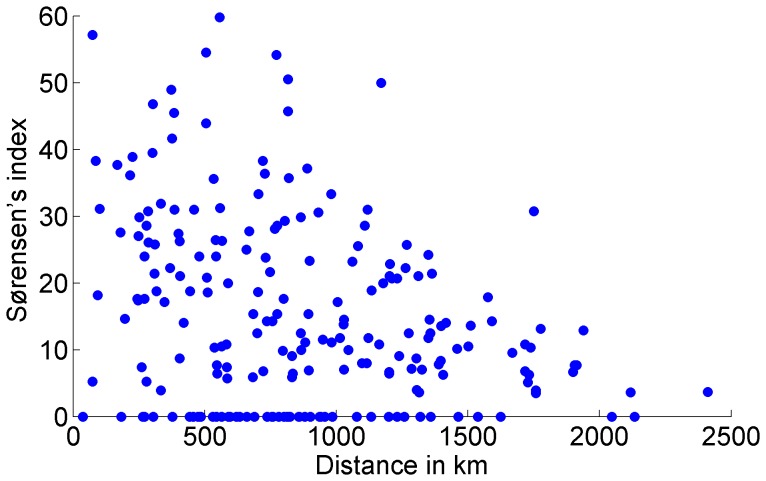
Sørensen similarity plotted against distance for all pairwise comparisons between drainage basins.

Figure 4 represents the calculated value of Sørensen’s index as a function of distance between all pairs of basins. The linear fit shows an inverse relationship between compositional similarity and distance, meaning that the similarity between the helminth faunas of the basins decays with the increasing distance between them.

## Discussion

The analyses in this work show that South-eastern Mexico exhibits a comparatively higher richness of neotropical helminth species, with some hydrological systems, such as Tabasco, Yucatan, Chiapas-Usumacinta and Papaloapan two to nine times richer than the northern, nearctic Durango, Rio Bravo and Río Tuxpan river basins. Given the general pattern of higher richness in the tropics, it is not surprising that the component of neotropical species is much greater than the nearctic species component (119 vs. 48). However, it is worth noting that the sampling effort has been different for each of the Mexican biogeographical provinces. The tropics have been studied far more intensively, with a greater number of basins explored, more frequency in sampling, and more fish families and species sampled [2], [7]. However, data suggests that in fact the pattern of increased richness in tropical environments is also true in the case of helminth parasites of freshwater fishes of Mexico. A greater richness in Mexican neotropical basins is possibly related to a greater ichthyological richness, and to the presence of fish families that harbour the richest communities of helminth parasites as our data set shows (see also [21]). The ichthyological complexity of south-eastern Mexico’s hydrological basins is much larger than the basins of Central Mexico; for example, in the state of Chiapas, the Río Usumacinta and Río Grijalva basins harbour 111 species of fish, while the Lerma basin is inhabited by 52 species. Fish species recorded from the Río Pánuco basin amount to at least 80 described native species, while in the Río Yaqui basin 51 species and in the Río Conchos (affluent of the Río Bravo) basin 53 species of freshwater fishes have been recorded [38]. Moreover, Poulin et *al*. [22] argue that a positive relationship between host and parasite species richness is generally supported and that this relationship is independent of variability among areas in terms of their size or in terms of sampling effort. Hotspots of host diversity are thus generally hotspots of parasite diversity too [22]. Tabasco, Papaloapan, Chiapas-Usumacinta and Yucatan Peninsula river basins are located geographically in the Mesoamerican hotspot [39], so it is possible to relate the richness of helminth parasites to the ichthyological composition of the basin [20], [25], [40], [41], [42]. The families of fish with high helminth richness like cichlids, characids and heptapterids are common and abundant in south-eastern Mexico, while the goodeids, relatively poor in helminths, are distributed in nearctic Mexico. Even though, cichlids and characids may be present in nearctic bodies of water, parasite richness is already diminished since these basins represent the limits of its distributional range. We can therefore suggest that the ichthyological composition of the basin can be used as a predictor of the distribution of the helminths, and is in fact an important determinant of the richness of the helminth fauna.

Our analyses allowed the distinction of Neotropical, Nearctic and Pacific Mexican drainage basins. Fish parasite fauna shows that half (11/21) of the Mexican basins analysed in this work classify as Neotropical. The close relationship between the helminths of Chiapas-Usumacinta and Tabasco hydrological systems was expected as most of these records belong indeed to the Río Usumacinta drainage basin, sampled form its middle part in Chiapas and from its lower part in Tabasco. The bodies of water sampled from the Yucatán Peninsula appeared as the area most associated with them, followed by the Río Papaloapan basin. This is further confirmed as distance between basins is taken into account: geographical proximity of the basins would allow frequent contacts and exchange of parasites that would explain high values of similarity between neighbouring basins. These similarities in helminth fauna are in accordance to the concept of the Usumacinta Ichthyological Province proposed by Miller [43]. This province extends from the Río Papaloapan in southeast Mexico, to the Polochic River in Guatemala. Furthermore, our data gives support to the proposal of Aguilar-Aguilar *et al.* [5], who consider that this area probably runs continuously from the Papaloapan river basin (including Los Tuxtlas, the Tabasco coastal plain and north-east Chiapas) to the Yucatán Peninsula. Tabasco along with the Papaloapan river basins are considered part of the Gulf of Mexico biogeographical province, while Yucatán is considered a somewhat more independent province [44].

In the same way, the close similarities observed between the second group of neotropical basins (Río La Antigua, Los Chimalapas and Río Tehuantepec) can be explained by the helminthological and the ichthyological composition of the faunas and by the geographical proximity of the basins. All these data confirm that parasite communities show decay in compositional similarity as a function of distance separating them. As shown by our results nearby host populations tend to have many parasite species in common, whereas distant ones share few [22], [45], [46].

The Balsas and Pánuco river basins as well as the Ayuquila and Santiago river basins have a mixture of nearctic and neotropical helminth species associated with the fish families that inhabit them. The presence of the ictalurid host *Ictalurus balsanus* and the goodeid *Ilyodon whitei*, in the Río Balsas basin, coupled with the record of three North American generalist helminths, provide a component of nearctic species in this basin. This is counterbalanced by several neotropical species of helminths associated to the cichlid *Cichlasoma istlanum* and the characid *Astyanax aeneus*. The neotropical species component is even larger in the three remaining basins mentioned (Pánuco, Ayuquila and Santiago), and is responsible for the strong similarity between these basins, while the presence of an ictalurid species in each basin contributes to the nearctic component. As for the main two neotropical groups of helminths discussed previously, the composition of the helminthofauna in the Balsas, Pánuco, Ayuquila and Santiago basins is explained because the geographical distribution of the helminth fauna reflects the distribution of the ichthyological fauna. Traditionally, the Lerma and Santiago basins have been considered as a single biogeographic unit for different taxa particularly freshwater fishes [40], [41], [47]. However, our analysis supports previous studies by Aguilar-Aguilar et al. [3], [4] who showed that these rivers do not form a single biogeographic unit.

The helminth composition and the position in the multivariate analyses of the bodies of water of the Cuatro Ciénegas Valley, geographically located in the Nearctic realm, can be explained by the fact that only cichlids and characids have been examined in this area, and that these are essentially neotropical fish families [19], [20], [48], [49]. Similarly, the only records from the Tuxpan river belong to helminths of the channel catfish, *I. punctatus* (Ictaluridae) [50] a nearctic species. This explains the close relationship between the Río Bravo and Río Tuxpan (although geographically located south of the Río Pánuco basin) as nearctic basins. The close relationship between the Río Lerma basin and the bodies of water of Durango, both located in the nearctic region may arise because, as previously suggested [3], [4], [44], [51] their taxa could share a common biogeographic history. Historical factors play indeed a very important role regarding the diversity of this area. The aridification of Northern Mexico in the past 10000 years is a possible explanation of the lower levels of diversity in the nearctic province [44], [52]. Similarity between the Lerma and Durango basins could be explained by the existence of ancient geological connections; the existence of a single paleolake on the Central Highland Plateau and its posterior fragmentation by extensive vulcanism during the Miocene and Pleistocene is well documented [53], [54], [55], [56].

The third set of basins, the Pacific affinities group, includes the oases of Baja California Sur, rivers and streams near Chamela Jalisco, and bodies of water belonging to either, the Río Papagayo or Río Atoyac basins. This group can be explained by the distribution of the basins along the Pacific versant of Mexico, by the geographic proximity between the Papagayo and Atoyac basins and between the Baja California and Ayuquila basins and also by the way their fish hosts families and their parasites disperse. The records of helminths in these basins are primarily associated with eleotrids and a species of mullet *Agonostomus monticola*, which migrate along the coast, entering continental bodies of water from brackish environments. The absence of these eleotrids and mugilids from nearctic environments results in a strong differentiation of this set of basins compared to the Pacific ones. These Pacific basins can also be differentiated from the neotropical ones by the peripheral distribution and the number of species of eleotrids and mugilids, given the fact that species of either families are rarely examined in more inland neotropical basins.

What this paper shows is that the helminth fauna of freshwater fishes of Mexico can characterise hydrological basins the same way as the fish families do. The data presented above show that the basins of south-eastern Mexico harbour a predominantly Neotropical helminth fauna whereas the basins of the Mexican Highland Plateau and the Nearctic area of Mexico harbour Nearctic fauna, following the same pattern of distribution of fish host families. The analysis shows that the composition of the helminth fauna of each particular basin depends on the structure of the fish community rather than on the limnological characteristics and geographical position of the basin itself and that the similarity decreases with increasing distance between drainage basins, the same that holds for their host families [38], [43]. The patterns of distribution of helminth species could be more related to the ancient geology of the basins, which explains the distribution of fish that inhabit them, than to the modern hydrological characteristics of each basin. However, it is interesting to acknowledge that the challenge for parasite species is given by the fact that their life cycles are complex and that to colonise a given environment they require not only the presence of the definitive host, but also the presence of suitable intermediate hosts. Following the arguments given by Salgado-Maldonado [19], this could be explained for example, for Camallanidae and Rhabdochonidae, the nematode families with the largest number of known species in Mexico, because they use broadly distributed and abundant copepods or aquatic insect larvae as intermediate hosts. A number of trematode lineages, on the other hand, use a single species of mollusc broadly distributed and abundant in some locations as their intermediate host.

## Supporting Information

Figure S1 **Dendrogram resulting from the similarity matrix based on the Sørensen Similarity Index for all river basins with introduced species.** Groups are based on parasite species composition of the basin.
(TIF)

Figure S2 **Non Metric multidimensional scaling (nMDS) ordination plot resulting from the resemblance matrix of the river basins, based on the Sørensen Similarity Index, with introduced species.**
(TIF)

Table S1 Most widespread species helminths of freshwater fishes of Mexico.
(DOCX)
