# Supplementary figures and images for: Patterns of Distribution of the Helminth Parasites of Freshwater Fishes of Mexico
Source: PLoS One. 2013 Jan 24;8(1):e54787. doi: 10.1371/journal.pone.0054787 (PMC3554625; doi:10.1371/journal.pone.0054787)

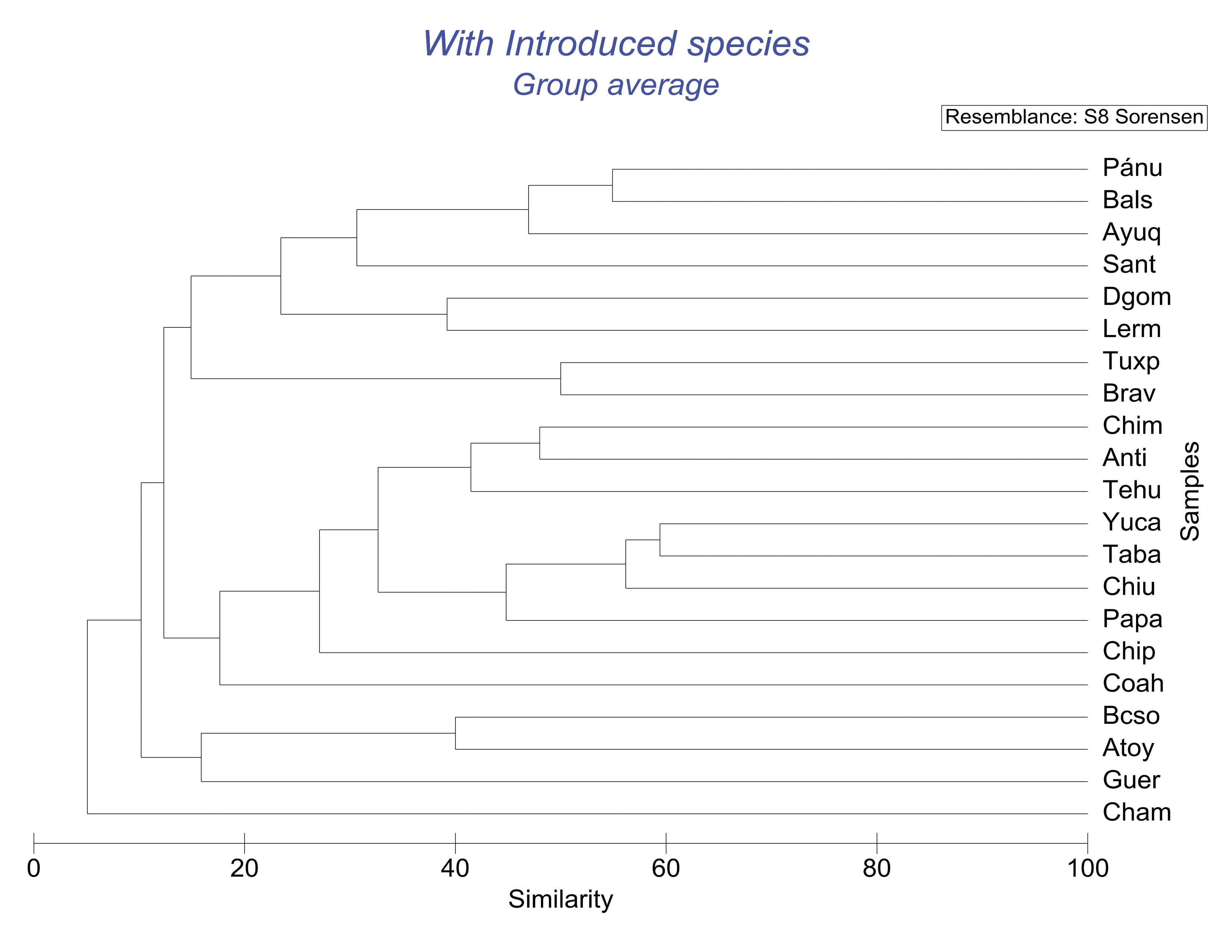

Supplement: Figure S1 — Dendrogram resulting from the similarity matrix based on the Sørensen Similarity Index for all river basins with introduced species. Groups are based on parasite species composition of the basin. (TIF) [file pone.0054787.s001.tif]

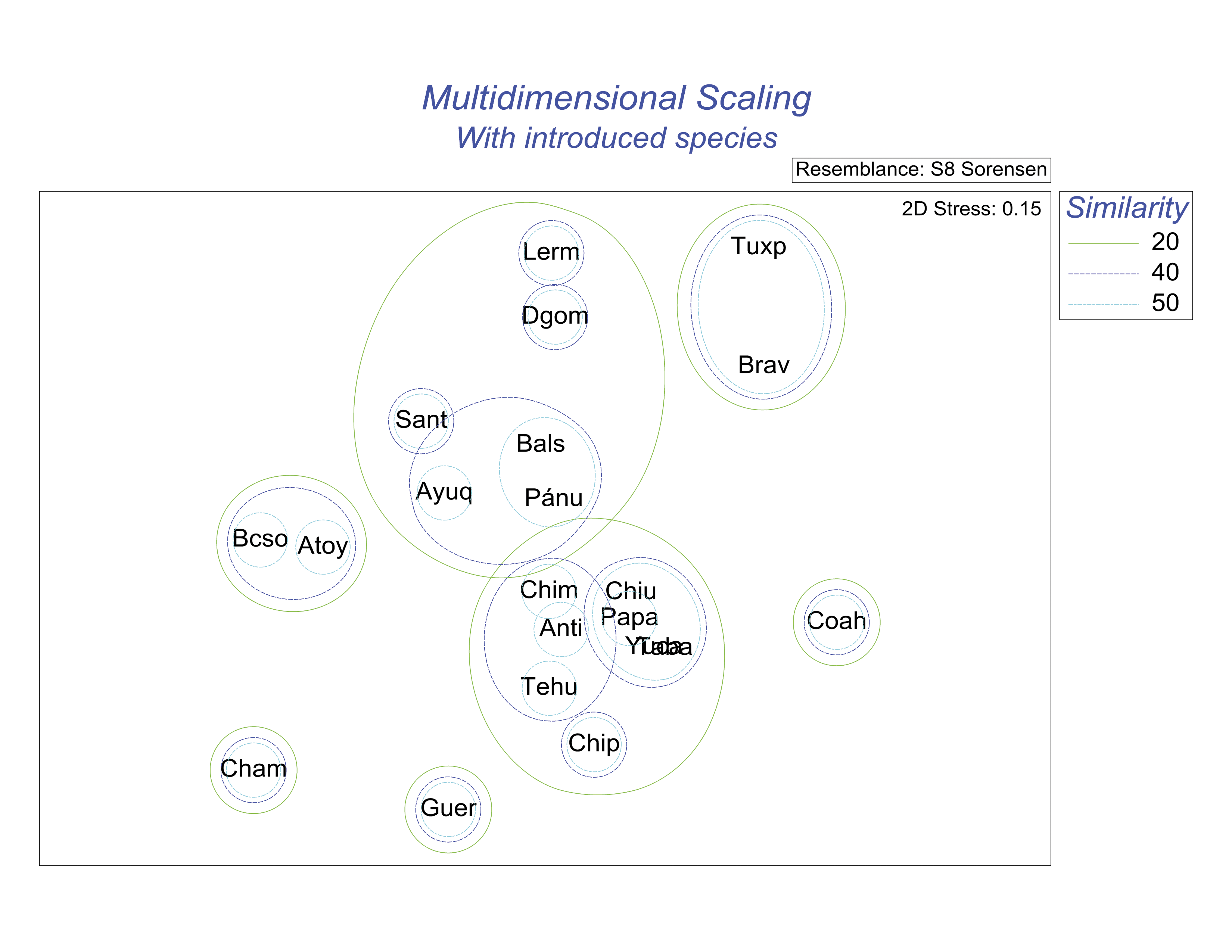

Supplement: Figure S2 — Non Metric multidimensional scaling (nMDS) ordination plot resulting from the resemblance matrix of the river basins, based on the Sørensen Similarity Index, with introduced species. (TIF) [file pone.0054787.s002.tif]
