# Supplementary material for: Patterns of Distribution of the Helminth Parasites of Freshwater Fishes of Mexico
Source: PLoS One. 2013 Jan 24;8(1):e54787. doi: 10.1371/journal.pone.0054787 (PMC3554625; doi:10.1371/journal.pone.0054787)
Supplement: Table S1 — Most widespread species helminths of freshwater fishes of Mexico. (DOCX) [file pone.0054787.s003.docx]

|  | Number of Basins |
| --- | --- |
| *Bothriocephalus acheilognathi* Yamaguti, 1934 | 12 |
| *Crassicutis cichlasomae* Manter, 1936 | 10 |
| *Saccocoelioides* sp. (ex.: Poeciliidae) | 10 |
| *Rhabdochona kidderi* Pearse, 1936 | 10 |
| *Magnivitellinum simplex* Kloss, 1966 | 9 |
| *Gyrodactylus* sp. 2 (ex.: Poeciliidae) | 9 |
| *Culuwiya cichlidorum* Aguirre-Macedo and Scholz, 2005 | 8 |
| *Genarchella isabellae* (Lamothe-Argumedo, 1977) Kohn, Fernandes, Gibson and Fróes, 1990 | 7 |
| *Aphanoblastella travassosi* (Price, 1938) Kritsky, Mendoza-Franco and Scholz, 2000 | 7 |
| *Urocleidoides strombicirrus* (Price and Bussing, 1967) Kristsky and Thatcher, 1974 | 7 |
| *Procamallanus* (*Spirocamallanus*) *rebecae* (Andrade-Salas, Pineda-López and García- Magaña, 1994) | 7 |
| *Rhabdochona mexicana* Caspeta-Mandujano, Moravec and Salgado-Maldonado, 2000 | 7 |
| *Sciadicleithrum bravohollisae* Kritsky, Vidal-Martínez and Rodríguez-Canul, 1994 | 6 |
| *Characithecium costaricensis* (Price and Bussing, 1967) Mendoza-Franco, Reina and Torchin, 2009 | 6 |
| *Neoechinorhynchus golvani* Salgado-Maldonado, 1978 | 6 |
| *Procamallanus* (*Spirocamallanus*) *neocaballeroi* (Caballero-Deloya, 1977) | 6 |
| *Paracapillaria teixeirafreitasi teixeirafreitasi* (Caballero-Rodríguez, 1971) | 6 |
| *Dichelyne mexicanus* Caspeta-Mandujano, Moravec and Salgado-Maldonado, 1999 | 6 |
| *Creptotrema agonostomi* Salgado-Maldonado, Cabañas-Carranza and Caspeta-Mandujano, 1998 | 5 |
| *Genarchella astyanactis* (Watson, 1976) Scholz, Vargas-Vazquez and Salgado-Maldonado, 1995 | 5 |
